# The medical school dean: understanding the development of a leader through self-determination theory

**DOI:** 10.1108/LHS-10-2025-0175

**Published:** 2026-04-17

**Authors:** Lulu Alwazzan

**Affiliations:** Department of Medical Education, College of Medicine, Imam Mohammad Ibn Saud Islamic University, Riyadh, Saudi Arabia

**Keywords:** Leadership, Leader, Management, Organizational culture, Leadership development, Medical education, Academic medicine, Medical school

## Abstract

**Purpose:**

This study aims to explore how medical school deans develop as leaders within their institutional context, focusing on how they acquire decision-making capacity, exercise influence and sustain their roles in administration. Using Self-Determination Theory (SDT) as a lens, the study examines key components of leadership development, including autonomy, competence and relatedness.

**Design/methodology/approach:**

A qualitative study design was employed using semi-structured interviews with medical school deans in Riyadh, Saudi Arabia. Participants were recruited via e-mail and provided informed consent prior to participation. Interviews were conducted virtually, audio-recorded and transcribed verbatim. Data were analyzed using framework analysis to identify themes aligned with the constructs of SDT.

**Findings:**

Eight themes were identified across the three SDT domains. Autonomy was reflected in defining autonomy and role negotiation. Competence in leadership emerged through experiences of mastery, the influence of resources, participation in formal leadership programs and achievement of organizational goals. Relatedness was reflected in interpersonal relationships and personal fulfillment. These findings illustrate that leadership development is shaped by both individual experiences and institutional contexts.

**Research limitations/implications:**

Although, this study sheds light on the complex interplay between leaders’ autonomy, motivation to lead and leaders’ ability to motivate others and how that contributes to organizational culture, a larger sample size, which includes deans from other Saudi Arabian cities, is needed for the findings to be transferable to other contexts. Furthermore, there was a lack of investigator triangulation. Time and access to the resources available for this research were constrained, both in terms of time and funding. As a result, the decision was made to streamline the analysis process to ensure the completion of the study within the specified constraints. Moreover, although the study shows a positive impression of leadership training programs, it does not measure actual outcomes or impact. As a result, the full effect of educational programs formal or informal cannot be determined.

**Practical implications:**

Despite the methodological limitations of this study, the findings have several implications for educational practice and policy. First, at the individual level, leaders and candidates for administrative positions should be educated in their leadership roles. Leaders need to understand what role autonomy, competency and relatedness play in their development as leaders and how to best cultivate these aspects while practicing leadership. Second, in terms of the organizational level, policies regarding leadership recruitment and development of leaders belonging to the medical school need to be developed. Such policies may foster a better workplace culture that prioritizes leadership development, which may lead to efficient and effective organizations.

**Social implications:**

This study highlights how leadership development in academic medicine extends beyond individual skill-building to social and organizational transformation. By viewing deans as learners embedded within relational, cultural and institutional contexts, it underscores that autonomy, competence and relatedness are socially constructed and sustained. Supporting these needs can improve workplace culture, reduce burnout and enhance collaboration among academic leaders. The findings suggest that fostering connected, motivated leadership in medical schools contributes not only to individual well-being but also to institutional resilience and the broader societal goal of developing effective, ethical and community-oriented health-care education systems.

**Originality/value:**

This study contributes to the limited literature on leadership development among medical school deans by applying a theoretical lens to understand their lived experiences. It highlights that leadership development extends beyond technical skill acquisition and involves a dynamic process of negotiation autonomy, building competence and fostering meaningful professional relationships. The findings offer insights for designing contextually relevant leadership development initiatives in medical education.

## Background

Leadership research in the field of medical education remains central, mostly because of the ever-evolving responsibilities for medical school deans and the accountability-driven context they work in Hitt and Tucker (2016), Leithwood (2001) and Muijs (2011). As a result, evidence-based practice in medical education leadership is crucial. The management of medical schools is of vital importance because of the massive contribution of medical schools to the health-care workforce. The literature emphasizes the impact of leadership on the effectiveness of medical schools and undergraduate and postgraduate medical education quality (McKimm and Lieff, 2021; Lieff, 2009). Deans have a considerable potential in creating environments for faculty and learners that encourage excellence in education, scientific research production and translation and community service (Lieff *et al.*, 2012; Lieff and Yammarino, 2017). Research resulted in numerous works on leadership styles (Steinert *et al.*, 2012), leadership identity formation (Lieff *et al.*, 2012) and leadership development (Alwazzan and Al-Angari, 2020). Less research is conducted on the *leader-as-learner* and how a leader learns to be a leader in their sociocultural context (Kaur and Noman, 2020).

Leader and leadership development are essential. Professionals take on formal leadership positions in medical schools, such as deans, vice-deans, heads of departments and program directors based on interest and career ambitions (Lieff *et al.*, 2012). While previous research explored the development of skills and styles among leaders (Lieff, 2009), we do not fully know how they go about enacting their leadership role while also learning about leadership. Here, we draw a distinction between *the development of a leader* and *leadership development*. The former is concerned with the individual’s *ability* to lead, while the latter focuses on a *process* of development that involves and influences multiple people simultaneously (Day, 2000). In this study, attention is paid to these two concepts, not in how they are different but in how they are related.

Individual leadership competencies can be developed over time (Ashford and DeRue, 2012). They can develop through mentoring, focused reflection and personal development (Ashford and DeRue, 2012), challenging assignments (Day and Dragoni, 2015) and formal leadership development programs (Alwazzan and Al-Angari, 2020). Most likely, a combination of these educational approaches can be used to prepare candidates and teams for their leadership roles (Pololi *et al.*, 2009; Pololi and Evans, 2015; O'Neil, 2008; Sherk *et al.*, 2009). To be mentored and to receive challenging assignments is not done individually but requires others, both seniors and juniors, to play a role. Therefore, *leader-as-learner* is then not an individual undertaking but a relational and organizational process.

In the literature, leadership has been extensively examined through multiple theoretical/conceptual frameworks. Classical trait perspectives focused on individual characteristics and behaviors. On the other hand, contingency theories gave special consideration to situational context to shape leadership effectiveness (Northouse, 2019; Fiedler, 1964). More recent leadership theories such as authentic and transformational leadership, emphasize values and influencing others, ultimately shaping organizational culture (Avolio and Gardner, 2005; Bass and Riggio, 2006). Health care and higher education contexts may be considered complex requiring more nuanced frameworks like the distributed leadership framework, this framework the interdependence and the need for collective responsibility (Spillane, 2005; Gronn, 2002). Collectively, the models highlight that leadership is not solely positional but an interpersonal process that is shaped by context.

In medical education, the complexity is increased because institutes may deliver education and research while simultaneously delivering clinical services. Previous works in medical education examined leadership practices (Frich *et al.*, 2015), organizational culture (Mann, 2011) and leadership development in varying specialties (Straus *et al.*, 2013). However, much of the produced research has focused on leadership behaviors and reporting leadership development interventional programs. Less has been published on how individuals develop leadership identities.

Progression of careers into leadership positions and role in academic medicine was also explored, demonstrating that leadership trajectories are not always linear and highly influenced by role expansion rather than deliberate career planning (Frich *et al.*, 2015). Aspirations for leadership are shaped by perceived self-efficacy as well as feasibility, that is, how one balances leadership responsibilities with other professional and personal obligations (Mann, 2011). These qualities are highly important for medical school deans because deanship carries a high level of accountability and role ambiguity.

Formal as well as informal processes of leadership development have been studied in academic medicine. Formal approaches include structured leadership programs, executive education and fellowships aimed at developing leadership competencies. Such competencies include strategy building and execution, interpersonal skills and conflict management (Straus *et al.*, 2013). Informal development happens through experiential learning, mentoring and peer communities of practice (Sambunjak *et al.*, 2006; Wenger, 1998). Evidence suggests that the combination of formal and informal leadership approaches embedded within work contexts is most effective (Day, 2000). It is important to note that the availability of leadership development programs across institutions is variable and often costly. As a result, not everyone has access to such opportunities (Straus *et al.*, 2013).

Fewer studies have conceptualized leadership development as an internal process, especially in medical education. Understanding why faculty members aspire to leadership and how they remain engaged is worthy of exploration. Self-Determination Theory (SDT) offers a framework by which we can examine leader development through core psychological needs: autonomy, competence and relatedness (Ryan and Deci, 2000). Examining leadership development through an SDT lens takes us beyond skills and behaviors, but a learner-centered and context-dependent process shaped by autonomy and personal agency, competence and growing mastery, and professional relationships and relatedness.

## Gap in the literature, study aim and research questions

Although a wealth of leadership literature exists in medical education, to the knowledge of the author, none has explored the individual development of leaders through an SDT lens. The latter will enrich our understanding of leadership at the individual granular level. SDT is a theory of human motivation and development that posits three psychological needs: autonomy, competence and relatedness as essential for sustained motivation and learning. This theoretical lens was selected for the study because it provides a lens for understanding how individuals internalize their leadership roles and not only how they acquire managerial skills. Moreover, although many studies have addressed leadership development by cataloging the experiences of leaders, in this study, the exploration of *leader-as-learner* is explored. As a result, our understanding of leadership development and how it should be addressed is deepened.

In this study, the constructs of autonomy, competence and relatedness from SDT are drawn upon. They are defined as core psychological needs that shape leadership development and for the purposes of this study. Autonomy refers to the experiences of agency and self-endorsements in leadership role enactment. Competence is about the perception of effectiveness and mastery of leadership skills. Finally, relatedness refers to the experiences of connection and belonging one experiences within professional settings. These constructs were used as guides rather than predetermined categories imposed on participants. The following research questions were developed to explore how medical school deans self-describe and interpret their experience of autonomy, competence and relatedness in their leadership development:

*RQ1*. How do medical deans develop autonomy in their leadership?

*RQ2*. How do medical deans perceive competency in their leadership?

*RQ3*. How do medical deans experience relatedness in medical schools?

## Theoretical framework

To orient this study, it is important to draw on a theoretical framework to organize thinking around the phenomenon at hand (Deci and Ryan, 2008). SDT was chosen to guide this study because it is a well-established, empirically supported argument of the process by which individuals develop and maintain motivation, as well as internalize complex organizational roles such as the role of a leader. Opposite other leadership theories which focus on traits, styles or behaviors, SDT roots the leader as a *learner-in-context*. This makes the theory suitable for understanding how medical school deans come to develop as leaders and enact leadership roles over a period. The latter is the main objective of this, that is, not only how deans develop as leaders but also how they find meaning in their leadership (Deci and Ryan, 2008; Ten Cate, 2017; Kusurkar *et al.*, 2011). This theory has a set of psychological mechanisms relating to the self, mainly that humans are growth-oriented, who are inclined to develop and to do so within and in coherence with the larger social structure (Rees *et al.*, 2023). This growth can be encouraged or discouraged by internal or external factors. To continue to grow, there is a need for autonomy, competence and relatedness to others in the larger social context (Ten Cate *et al.*, 2009).

In SDT, a great emphasis is put on motivation to grow, such motivation can range from lack of motivation (amotivation), internal motivation and external motivation. External motivation can lead to internalization of certain values. To remain internally motivated, the need for autonomy, competence and relatedness must be satisfied. A high internal motivation, for example, aspiration for leadership leads to better understanding of leadership processes and higher satisfaction with the leadership experience (Bartholomew *et al.*, 2011; Deci and Ryan, 2012).

## Materials and methods

### Study design

The study is underpinned by social constructionism, whereby individuals construct and interpret realities based on their own experiences and interplay with others and the environment (Edward *et al.*, 2000). Social constructionism views knowledge and meaning as generated through social interaction and social context. As a result, leadership development is understood as a process that is socially constructed influenced by relationships, institutional roles and cultural expectations, rather than a group of traits. To that end, a qualitative research approach was used for this study, using narrative inquiry, asking participants open-ended questions such as *what does this concept mean to you? How did it influence you?* The latter approach encourages understanding the human experience through the construction and reconstruction of narratives (Deci and Ryan, 2000; Crotty, 2003) thus affording the researcher an opportunity to understand the complexities of leader and leadership development particularly in relation to motivation, meaning-making and psychological need satisfaction The study was conducted in accordance with the guidelines of the Declaration of Helsinki and approved by the Institutional Review Board of the College of Medicine, Imam Mohammad Ibn Saud Islamic University.

### Context

In the Kingdom of Saudi Arabia, the landscape of medical education has undergone great transformation. Over the course of a decade, the number of medical schools surged from 5 institutions using traditional discipline-based curricula to over 40 medical schools featuring diverse curricular approaches. These approaches span the spectrum from traditional models to more progressive, problem-based and community-oriented programs. In addition to human capital, medical schools in Saudi Arabia provide medical services to society and produce scientific research. Therefore, medical school deans are expected to lead complex systems that hold a large responsibility. Medical schools can be categorized into governmental and private schools. Schools included in this study may be categorized into urban city-based schools. The schools vary in size, serving a student body of 500–2000 undergraduate medical students and use 150–400 faculty members in varying specialties, including clinical and basic science faculty. These schools operate within teaching hospital networks, as urban-based institutions, they function within highly regulated national accreditation standards and are embedded in the broader context of Saudi Arabia’s Health Transformation Program and Vision 2030 initiatives. Deans in these contexts typically oversee complex organizations that integrate training, clinical practice and research agendas.

### Setting, data collection and participants

The study was conducted in Riyadh, Saudi Arabia during the months of July–May 2022. It included two private school deans and three governmental school deans. Data were collected by semi-structured individual interviews. Interviews were expected to produce rich in-depth data. They were found appropriate because this study is concerned with meaning and does not aim to generalize the findings (Riessman and Quinney, 2005; Alwazzan, 2023; Crouch and McKenzie, 2006). To be eligible for participation, participants must have at least one year of work experience as a dean. Given access to resources and time, the study was limited to the capital city Riyadh where there are currently eight medical schools. In accordance with contemporary methodological practice, the aim of this qualitative study is to elicit rich, reflective accounts from participants’ leadership experiences. Generalizability and increased sample size were not an aim (Varpio *et al.*, 2017).

The researcher contacted potential participants via e-mail invitation giving an overview of the research, eligibility criteria and contact details of the researcher. To ensure the anonymity of those who wish to participate, they were asked to contact the researcher directly. The researcher then proceeded to personally contact the participants by phone, give an overview of the research and arrange for the interview. Interviews were 30–60 min long and were conducted in English. They were audio-recorded and each recording was given a unique identifier to protect the identity of the participant and stored in a specific drive for the project. The semi-structured interviews were guided by an interview guide (Supplementary Material 1) developed by the researcher based on the chosen theoretical framework and the literature review. Consent was sought at the beginning of the interview and participants were asked for verbal and written consent. Participants were informed of their anonymity and right to withdraw at any time.

### Data analysis

The portfolio of data generated was secured in three electronic folders. A folder for the interview transcripts, the text and its analysis and the text translations. Quotes included in the article were translated from Arabic to English by the author (then back translated by a translator to ensure consistency) for the purpose of publication. Data from the text and interview transcripts were analyzed using framework analysis. Framework analysis is defined as “an analytical process which involves a number of distinct though highly interconnected stages” (Ritchie and Spencer, 1994) (p. 177). The framework has five phases of analysis including: familiarization, identifying a thematic framework, indexing, charting and mapping/interpretation. Following initial coding and theme development by the author, a second reviewer with experience in qualitative research independently reviewed a subset of the audio transcripts, as well as the developed themes and definitions. The two reviewers met to discuss their independent findings. This process was done several times until both reviewers agreed on the final analysis. This iterative process increased analytic rigor.

## Results

Five out of eight deans working in the capital Riyadh, Saudi Arabia took part in this study. Deans’ ages ranged from 42 to 53 years, the sample included both male and female participants, years served as a dean ranged 1–10 years (see Table 1 for participant details)

**Table 1.** Participant demographics details

Participant ID	Gender	Years in leadership role (years)	Institutional context	Specialty
P1	Male	4	Public Medical School	Family medicine
P2	Male	1	Public Medical School	Pediatrics
P3	Male	5	Public Medical School	Pediatrics
P4	Male	10	Private Medical School	Surgery
P5	Female	2	Public Medical School	Ophthalmology

Eight themes were identified, two themes relating to *RQ1*: defining autonomy and role negotiation; four themes relating to *RQ2*: experience of mastery, influence of resources, formal leadership programs and evidence of realizing organizational goals; and two themes relating to RQ3: interpersonal relationships and personal fulfillment (see Table 2 for theme definitions and mapping). Each theme is presented in the following sections and illustrative quotes are shared.

**Table 2.** Theme definitions and mapping

SDT construct	Theme	Definition	What the theme captures
Autonomy	Defining autonomy	Participants’ understanding of autonomy as the capacity to exercise independent judgment, make evidence-based decisions and pursue leadership goals aligned with personal and institutional values	Agency, decision-making authority, internal motivation and value-driven leadership
Autonomy	Role negotiation	How participants negotiated competing personal and professional identities, including tensions between clinician, academic and leader roles, and between professional responsibilities and personal life	Internalization of leadership role, work–life balance and identity transition
Competence	Experience of mastery	Participants’ perceptions of their developing leadership skills, confidence and ability to manage complex organizational responsibilities	Self-efficacy, learning through experience, emotional regulation and coping with leadership challenges
Competence	Influence of resources	The impact of institutional support, material resources and human capital on participants’ ability to perform effectively as leaders	Structural enablers and constraints shaping leadership performance and motivation
Competence	Formal leadership programs	Experiences of structured leadership training as a source of learning, legitimacy and professional growth in leadership roles	Skill development, professional identity as a leader and preparation for leadership responsibilities
Competence	Evidence of realizing organizational goals	Participants’ reflections on achieving institutional outcomes and using performance indicators as markers of leadership success	Competence demonstrated through results, productivity and organizational impact
Relatedness	Interpersonal relationships	Participants’ experiences of connection, trust and reciprocity with colleagues, teams and senior leadership within the medical school	Belonging, social support, teamwork and relational leadership
Relatedness	Personal fulfillment	Feelings of satisfaction, purpose and meaning derived from contributing to the institution, professional community and national transformation agenda	Emotional engagement, sense of belonging and alignment with collective mission (e.g. Vision 2030)

To increase depth, each theme is illustrated by multiple quotations from different participants. Quotes are presented as extended excerpts to show the substantive nature of the interview dialogue. Themes are categorizes according to the research questions of this study:

RQ1.
How do deans develop autonomy in their leadership?

## Defining autonomy

When asked “what is your understanding of autonomy in your leadership?”, participants conceptualized autonomy in three ways. First, autonomy related to decision-making is a necessary skill for the dean. This was viewed as the individual’s ability to decide on one’s own. This is influenced by the magnitude of the decision that needs to be made, and if the dean had prior experience. The need for competence in decision-making refers to the sense of self-efficacy and belief in one’s ability to perform and make an impact. As the following participant shared:

My leadership shows in my ability to make difficult decisions and to be firm about it, the more experience I gain the further I can go with my decisions (Dean_1).

Another participant shared:

Having autonomy for me means being trusted by others to make decisions that affect their daily activities. At the beginning, I was cautious and dependent on my senior peers, consulting them on what I should do. In time, I became more confident in my judgment. Nowadays, I feel that my autonomy came from my experience over the years and knowing that my decisions are based on a rich leadership experience (Dean_2).

Second, participants view their autonomy as it relates to supporting a decision with scientific evidence. As practicing physicians, deans often believed their scientific training and commitment to evidence-based practice gave them more authority and enabled them as leaders, as one participant shared:

I’m highly trained with advanced degrees and I’m a highly published author in my field, my decisions are backed-up by my extensive training and experience. I don’t do things without reading extensively about it (Dean_2).

Another participant shared a similar account:

my clinical and academic background highly influences how I manage and lead. Each decision must be justified scientifically as far as I’m concerned. I want others to trust that my decisions are not my personal opinions, that I depend on benchmarks, data, guidelines and what is done worldwide (Dean_3).

These quotes illustrate how autonomy was experienced by deans as grounded in evidence and experience reinforming participants’ sense of self-efficacy and legitimacy as leaders.

Third, autonomy manifested as being self-determined to pursue goals and activities. Participants expressed a need for making decisions that are situated in the broader context, stating that medical schools are no longer seen as mere schools graduating practicing physicians, but as entities contributing to the health-care workforce and influencers of the health economy:

We are not producing doctors; we are contributing to the economy. If you think of it this way you recognize that as a leader you must have great ambitions and goals for the school (Dean_4).

## Role negotiation

Participants also negotiated their professional versus personal roles as well as in between their professional roles. Learner autonomy was often challenged by deans’ personal lives. Participants admitted to a compromise in one’s personal life to fulfill their leadership role as dean and in turn learn, as one participant shared:

To do this job I’ve sacrificed my personal life, my children must be more independent. Although I think there must be better models for work-life balance. I have colleagues whose solution is to leave the position to make time for their personal lives. I’m continuously looking for ways to balance, that is my responsibility as a leader to learn how to balance (Dean_1).

Another participant shared:

I was first a vice-dean of academic affairs then I became a dean, when that shift happened I realized I was no longer only a physician or a manager of affairs. My role changed dramatically, my time and priorities shifted, and I had to make important decisions on how to navigate my new role. This I found to be a constant process, I needed to constantly reflect on what adjustments I needed to do (Dean_4).

The previous quotes show how participants negotiated their roles and how that process shaped their autonomy. Role negotiation seems to be an active ongoing process requiring deans to construct meaning and seek balance.

Another role negotiation that was appreciated in the data was the one between different professional roles. That is, many participants spoke about their different professional roles, their roles as clinicians, teachers, researchers and leaders. One participant shared:

I don’t spend much time in the clinic now that I’m the dean. I think this job is demanding and you must be ready for it and there are sacrifices you need to make. That is a decision I made to grow professionally. I don’t view it as a bad thing (Dean_3).

RQ2.
How do medical deans perceive competence in their leadership development?

## Experience of mastery

This theme considered participants’ abilities and experiences of mastery. Most of the data from junior deans focused directly on the internal conflict, the regulation of self-motivation to lead and facing burnout. For example:

There are many demands. I’m overwhelmed most times, and I find difficulty in delegating to others (Dean_1).

Another participant shared:

In the beginning, I questioned my ability of managing everything and everyone. The workload was demanding at times and overwhelming. I struggle with delegation, I felt I had to control every detail. I’m very aware of quality, its important to me. I’ve since learned that my job was to instill in others the importance of quality, not to do it myself. I became more confident as a leader (Dean_3).

The quotes illustrate competence as a developmental process, where gradual learning takes place rather than being immediate.

Most participants spoke about situations where external parties influenced productivity and efficiency as a leader:

If we have support from senior leadership, we can achieve, if we don’t have the support, it is very difficult to accomplish anything (Dean_2).

Although some participants narrated situations where they were able to demonstrate competence, this demonstration was sometimes undermined by a lack of senior team members who provide a mentorship role, stating reticence in initiation of certain activities, as the following participant shared:

It is challenging to motivate everyone all the time. The vice-deans and the head of departments take on some of the responsibility for their own teams. I need a senior someone to motivate me too, to sign off on some of my plans. Some ideas, I leave because I’m thinking it is too small or too difficult (Dean_5).

Others shared the challenge in demonstrating competence in the face of different organizational challenges:

In our schools we remain optimistic no matter the situation, but optimism is not always beneficial. You either have the knowledge and skills to perform or you don’t. At the end of the day there are performance indicators that we must meet (Dean_4).

Another participant shared:

My ability to lead and my competency in leadership is constantly appraised, not just by myself but by the outcomes and KPIs I must deliver. This is a constant worry for me but also challenge where I can really grow and for my team to grow. This is especially true if my team is not the well experienced or we don’t have the enough resources (Dean_6).

These accounts show competence as performance oriented and constrained by the setting.

It was noticed that a senior leader talked less about the motivation to lead, whether internally or externally, rather on the ability to motivate others.

I don’t motivate myself, I motivate others, and others motivate me (Dean_2).

## Influence of resources

In this theme, participants spoke about the influence of resources on their competence such as the availability of material resources and the ability to recruit competent human capital. In terms of the availability of resources, one participant shared:

We are limited by resources; we don’t have enough to accomplish our desired goals. This demotivates me and those around me. In many ways I feel a responsibility to provide for my staff and their projects. As a leader who is constantly learning as I go, I find difficulty to thrive in a limiting environment (Dean_2).

In terms of human capital, one participant shared:

It is difficult nowadays to find people, competent people, who are willing to work in academics. Even when you are lucky to recruit them, it will be difficult to keep them in academics. This is one of the areas I need to develop in myself […] human capital management (Dean_3).

## Formal leadership programs

Participants spoke about leadership training programs as a way to introduce themselves as potential leaders to their professional community, as one participant shared:

I knew I wanted to be the dean, so leadership development programs were a way to announce that, that I’m working on myself improving my knowledge and skills formally (Dean_3).

Another participant shared:

Participating in formal programs showed different ways of looking at matters. It wasn’t only the skills but learning from others on what works in their contexts. It definitely gave me a space to reflect on my areas to be developed, I became more confident and more intentional about my growth (Dean_2).

Leadership programs operated for some as mechanisms of identity formation and not just sites of skill acquisition. Moreover, they increased the feeling of legitimacy in leadership.

Other participants spoke about leadership programs after the fact, serving as a catalyst for their growth, as one participant shared:

I became painfully aware of the skills I lacked when I took the job. As soon as a I realized, I looked around for workshops or something to orient me to my duties. There are some aspects I wasn’t aware of, financial management for example, you can find programs online (Dean_2).

## Evidence of realizing organizational outcomes

The accomplishment of outcomes was prevalent across all participant narratives. Leaders linked their motivation to work and the ability to motivate others to competence and the accomplishment of goals. For example:

Excellence is a choice. If you make that choice before you begin you will always be able to give fully and realize organizational goals (Dean_2).

However, participants often described their unpreparedness to deal with the volume of employees, as the following participant shared:

It is surprising to me that my teaching role did not end but took a different shape. Both senior and junior staff, need constant mentoring. I need a crash course on how to mentor many people, I think that affects our bottom line (Dean_5).

RQ3.
How do medical deans experience relatedness in medical schools?

## Interpersonal relationships

Participants spoke about the reciprocity among people when they felt a sense of connectedness and belonging. Positive interpersonal relationships nurtured a sense of security and caring:

I make an honest effort to get to know everyone I work with; I also make a point of inviting their families in reward ceremonies. I want them to celebrate their accomplishments with their family in addition to their professional family (Dean_5).

## Personal fulfillment

Participants put great emphasis on certain feelings being a driving factor of success, as individuals and as an organization: Personal fulfillment, engagement and quality of job performance were essential components of success:

I have my own ambitions for my development and career goals. My colleagues, and the university help me achieve them (Dean_4).

Another participant shared:

What motivates me in my leadership is knowing that I’m part of something bigger than myself. When I see faculty members and students grow and the medical school also grows and accomplishes organizational goals, I feel a deep sense of fulfillment (Dean_5).

Participants described job performance as directly related to a sense of belonging.

In my current role as dean I was able to connect with my vice-deans. I tried to build a team. They are my executive group. That makes me want to come to work that I’m supported by a leadership team (Dean_1).

Finally, participants often referred to the larger macro context and the national and health transformation taking place in Saudi Arabia as a reason for increased belonging:

What we do in the medical college in directly related to Saudi Vision 2030. We have a duty to realize the Health Transformation Program and we come to work every day with this in our minds, how to contribute to the greater good (Dean_5).

Another participant shared:

At the time of the country’s development gives me more meaning. I do not see my role as someone who leads a medical school but as someone who shepherds future doctors into the growing workforce. I really believe what I do with students matters beyond the walls of the school and for longer than I can fathom (Dean_2).

These quotes illustrate the relatedness to a broader level, where motivation is not only interpersonal but also alignment with national goals and collective identity.

## Discussion

This study takes a granular view of medical school deans’ development within the Saudi Arabian context, examining their perceptions through an SDT lens, emphasizing autonomy, competence and relatedness as central psychological processes shaping professional growth. When speaking of their autonomy as learners, participants of this study highlighted the ability to make a decision, being evidence-based in their leadership practice and being self-determined to pursue goals and activities as necessary skills. The results of this study show that deans prioritized leadership education and training, regarding it as a positive influencer and a reason for pursuing and remaining in leadership. To better our understanding of the role of autonomy in learning to be a leader, educators and researchers must expand their thinking beyond the role of formal leadership training; that is, formal training may be used to expand an individual’s leadership competency but through experiential learning and through managerial assignments that increase in difficulty as the leader grows, as mentioned in the literature (Day and Dragoni, 2015), may be a more effective means of learning. The themes of defining autonomy and role negotiation show how deans internalized leadership roles while balancing clinical, teaching and research responsibilities. These findings align with SDT’s construct of autonomy as a self-motivated action rather than mere independence. This underscores that leadership development involves meaning-making and alignment with personal values rather than mere positional authority.

Competence emerged as a process shaped by personal learning efforts as well as institutional support. Participant’s mastery of leadership reflected growing confidence and capability in managing complexity. Resources and formal leadership programs carry an important structural and educational support in fostering leadership effectiveness. The theme of realizing organizational goals shows that competence was experienced through realized outcomes. These findings suggest that leadership development is more sustainable when leaders perceive themselves as capable and supported in meeting institutional needs.

Relatedness was illustrated through interpersonal relationships and personal fulfillment because of contributing to the broader organizational mission. This underscores that leadership is a socially embedded process. Fulfillment is linked to feelings of belonging and alignment with national goals. This supports SDT’s principle that relatedness is essential to motivation.

These findings together demonstrate that leadership development among medical school deans can be understood as a process of psychological need rather than a set of generic leadership behaviors and skills. Drawing on the concept of relatedness, an interpersonal orientation to leadership is needed. Established leaders can be mentors, coaches or sponsors, providing career opportunities for potential leaders, guidance in navigating such opportunities and contributing to meaningful career growth. Fostering such professional relationships has been associated with greater career satisfaction (Pololi *et al.*, 2009; Pololi and Evans, 2015), thus increasing motivation for leadership and encouraging the development of self-regulation later. This leads to key considerations for the role of active professional relationship development and how such relationships can be fostered.

## Methodological strengths and limitations

This study is strengthened by its originality; to the author’s knowledge it is the first study using SDT to understand the development of leaders in academic medicine. The results are consistent with findings of other leadership development research. Although this study sheds light on the complex interplay between leaders’ autonomy, motivation to lead and leaders’ ability to motivate others and how that contributes to organizational culture, several limitations must be taken into consideration when interpreting the findings. First, the sample was based on convenience sampling and included a small number (*n* = 5). A larger sample size, which includes deans from other Saudi Arabian cities, is needed for the findings to be transferable to other contexts. Second, participants were drawn from a single city, which may limit the transferability of the findings to medical school deans working in other regions and health-care systems. Third, time and access to the resources available for this research were constrained, both in terms of time and funding. As a result, the decision was made to streamline the analysis process to ensure the completion of the study within the specified constraints. Moreover, although the study shows a positive impression of leadership training programs, it does not measure actual outcomes or impact. As a result, the full effect of educational programs formal or informal cannot be determined.

## Implications for educational practice and policy

Despite the methodological limitations of this study, the findings have several implications for educational practice and policy. First, at the individual level, leaders and candidates for administrative positions should be educated in their leadership roles. Leaders need to understand what role autonomy, competency and relatedness play in their development as leaders and how to best cultivate these aspects while practicing leadership. Second, in terms of the organizational level, policies regarding leadership recruitment and development of leaders belonging to the medical school need to be developed. Such policies may foster a better workplace culture that prioritizes leadership development, which may lead to efficient and effective organizations. The experiences described by those who took part in this study highlight that leadership ability within the dean role is not simply a function of seniority, but rather a developmental process shaped by institutional settings. As a result of this insight, specific areas where intentional intervention can ensure this developmental process are crucial. In addition to practice, the results of this study have implications for policy. There is a need for structured, system-level investments in leadership development with medical education. At a macro/policy level, findings of this study support the need for integration of formal leadership development frameworks into institutional strategies. Moreover, at a societal level, the study findings support fostering reflective and human-centered leadership cultures within medical education, encouraging institutions to recognize the individual and relational dimensions of leadership. Supporting leaders as a result can lead to improved healthier organizational cultures, where teachers and learners can thrive.

## Implications for research

Further qualitative and quantitative research is needed with expansion of the leader type such as heads of departments and program directors, to explore the generalizability of the findings beyond senior leadership (dean), as well as beyond the Saudi Arabian context. Further studies should aim to collect data from junior aspiring leaders in postgraduate training and who may have a different experience of leadership. Furthermore, researchers should evaluate the outcomes of interventions designed and implemented to improve leadership practice in medical education. As a result of this study, the use of SDT to understand the development of leaders in medical education and how this may inform the design of leadership development programs is recommended. Finally, this study shows that leadership is a relational process; therefore, more qualitative study designs are recommended to understand how professional relationships are initiated and nurtured over time.

## Conclusions

Leader and leadership development in Saudi Arabian medical education are vital for the growth of the health-care sector. Using an SDT lens, this study presented several findings that may direct future studies and educational leadership programs. Understanding the *leader-as-learner*, including the development of their autonomy, competency and sense of relatedness may help us better prepare deans and motivate them for their leadership roles in medical schools, contributing to the quality of medical education and practice.

## Supplementary Material

Data supplement 1

## Data Availability

The data presented in this study are available on request from the corresponding author. The data are not publicly available due to anonymity and protection of the participant privacy. The population of the study is limited, and participants may be easily identified.

## Supplementary materials

The following supporting information can be downloaded at: Supplementary Material A: interview guide.
