# Supplementary material for: The medical school dean: understanding the development of a leader through self-determination theory
Source: Leadersh Health Serv (Bradf Engl). 2026 Apr 17;39(5):49–65. doi: 10.1108/LHS-10-2025-0175 (PMC13094663; doi:10.1108/LHS-10-2025-0175)
Supplement: Data supplement 1 [file lhs-10-2025-0175_supplementary_file_1.docx]

The medical school dean: Understanding the development of a leader through Self-Determination Theory

**Interview guide**

**VERSION 1**

**English**

**Welcome**

• Welcome the participant

*“Welcome and thank you for agreeing to participate in this project. We recognize your busy*

*schedule and appreciate your cooperation.”*

**Introduction**

• Name and background

*“My name is Lulu Alwazzan and I'm an assistant professor at the college of medicine at Imam mohammad ibn Saudi Islamic University”*

• State the purpose of the interview

*“I’m conducting a study exploring leadership development amongst medical school deans including the emergence and enactment of leadership in medical education, as well as who is involved in giving shape to the leadership process in the Saudi Arabian context, you were nominated because your are a current or past dean of a medical school”*

**• Language**

*“The interview will be conducted in Arabic. However, feel free to express yourself in*

*English if you need to do so.”*

**• Queries**

*“Do you have any questions you would like to ask before we start?”*

**Anonymity and right to withdraw**

“• *The interview will be audio-recorded but your identity will remain anonymous.*

*• The audio files will be transcribed after the interview.*

*• You will be assigned a participant number to protect your identity.*

*• The audio files will be kept in a secure server abiding by research governance policies.*

*• You have the right to withdraw from the interview at any time with no penalty to yourself.*

*• You do not have to answer questions that make you feel uncomfortable. You may find some*

*of these issues personal. If this is the case, feel free to inform the researcher.*

*• Please refrain from mentioning specific dates, names of places or individuals.”*

| **Study objective** | **Research question** | **Interview questions** | **Prompts** |
| --- | --- | --- | --- |
| Explore medical deans’ perceptions of leadership autonomy | How do medical deans develop autonomy in their leadership? | Tell me about how your started in leadership? | Is it self-motivated? Nominated by someone else?  When did it start?  Where did it start? |
| Explore medical deans perceptions of leadership competency | How do medical deans perceive competency in their leadership? | Tell me about your competency as a dean? | Your style?  Your mentorship?  Working in teams?  Adopting a vision? |
| Investigate how deans experience belonging in medical schools. | How do medical deans experience relatedness in medical schools? | Tell me about how you your professional relationships as a dean? | How is your relationship with other employees?  Do you have a mentor?  Professional development programs? |

**Conclusion**

• Acknowledgment

*“Thank you for taking part in this interview. Your participation greatly enriched the study*

*and we appreciate your contribution”*

• Reiterate important information

*“After analysis of the data, we will send a copy of the report to everyone who requests to*

*receive one. Those will be given the opportunity to comment on the result before they are submitted for publication.”*

*“We assure your anonymity is of utmost importance throughout all stages of this study.”*
